# Supplementary material for: Gut microbiota diversity and composition in children with autism spectrum disorder: associations with symptom severity
Source: PeerJ. 2025 Jun 6;13:e19528. doi: 10.7717/peerj.19528 (PMC12147763; doi:10.7717/peerj.19528)
Supplement: Supplemental Information 5 [file peerj-13-19528-s005.docx]

**Table S1: The sequences informations of all samples**

| **Group** | **Sample_info** | **ASV_num** | **Seq_num** |
| --- | --- | --- | --- |
| **G1** | H_2 | 30 | 37247 |
|  | H_3 | 78 | 37247 |
|  | H_4 | 32 | 37247 |
|  | H_7 | 30 | 37247 |
|  | H_9 | 54 | 37247 |
|  | H_11 | 57 | 37247 |
|  | H_13 | 46 | 37247 |
|  | H_15 | 62 | 37247 |
|  | H_18 | 38 | 37247 |
|  | H_19 | 31 | 37247 |
|  | H_21 | 27 | 37247 |
|  | H_23 | 27 | 37247 |
|  | H_24 | 33 | 37247 |
|  | H_25 | 51 | 37247 |
|  | H_26 | 70 | 37247 |
|  | H_28 | 49 | 37247 |
| **Total** |  | 715 | 595952 |
| **G2** | C_2 | 44 | 37247 |
|  | C_4 | 21 | 37247 |
|  | C_5 | 57 | 37247 |
|  | C_6 | 48 | 37247 |
|  | C_8 | 98 | 37247 |
|  | C_26 | 37 | 37247 |
|  | C_27 | 49 | 37247 |
|  | C_28 | 40 | 37247 |
|  | C_29 | 40 | 37247 |
|  | C_30 | 33 | 37247 |
|  | C_31 | 55 | 37247 |
|  | C_33 | 23 | 37247 |
|  | C_35 | 47 | 37247 |
|  | C_36 | 43 | 37247 |
|  | C_37 | 31 | 37247 |
| **Total** |  | 666 | 558705 |
| **G3** | C_3 | 34 | 37247 |
|  | C_11 | 55 | 37247 |
|  | C_13 | 70 | 37247 |
|  | C_14 | 73 | 37247 |
|  | C_15 | 33 | 37247 |
|  | C_16 | 25 | 37247 |
|  | C_17 | 46 | 37247 |
|  | C_19 | 30 | 37247 |
|  | C_20 | 34 | 37247 |
|  | C_21 | 41 | 37247 |
|  | C_22 | 21 | 37247 |
|  | C_23 | 76 | 37247 |
|  | C_24 | 44 | 37247 |
|  | C_25 | 25 | 37247 |
|  | C_32 | 33 | 37247 |
| **Total** |  | 640 | 558705 |
